# ASO Author Reflections: Surgical Strategy for Perihilar Cholangiocarcinoma

**DOI:** 10.1245/s10434-024-15156-5

**Published:** 2024-03-06

**Authors:** Pim B. Olthof, Bas Groot Koerkamp, the Perihilar Cholangiocarcinoma Collaboration Group, L. Aldrighetti, R. Alikhanov, F. Bartsch, W. O. Bechstein, J. Bednarsch, C. Benzing, M. T. de Boer, S. A. Bouwense, S. Buettner, I. Capobianco, M. Cescon, R. Charco, M. I. D’Angelica, M. Dewulf, P. de Reuver, E. de Savornin Lohman, M. Efanov, J. I. Erdmann, L. C. Franken, J. Geers, M. C. Giglio, S. Gilg, C. Gomez-Gavara, A. Guglielmi, T. M. van Gulik, J. Hagendoorn, A. Hakeem, J. Heil, F. J. H. Hoogwater, J. N. M. IJzermans, H. Jansson, W. R. Jarnagin, G. Kazemier, T. P. Kingham, H. Lang, P. Lodge, S. K. Maithel, M. Malago, H. Z. Malik, R. Margies, R. Marino, Q. I. Molenaar, S. Nadalin, U. Neumann, T. A. Nguyen, L. E. Nooijen, C. L. M. Nota, S. W. M. Olde Damink, E. Poletto, R. J. Porte, R. Prasad, J. Pratschke, L. M. Quinn, F. Ratti, M. Ravaioli, K. J. Roberts, J. Rolinger, A. Ruzzenente, E. Schadde, M. Schmelzle, A. A. Schnitzbauer, M. Serenari, E. Sparrelid, A. Sultana, B. Topal, R. I. Troisi, S. van Laarhoven, B. M. Zonderhuis

**Affiliations:** 1 Department of Surgery, Erasmus Medical Center, Rotterdam, The Netherlands; 2 Department of Surgery, Amsterdam UMC, Amsterdam, The Netherlands; 3 Department of Surgery, University Medical Center, Groningen, Groningen, The Netherlands

## Past

The goal in the surgical treatment of perihilar cholangiocarcinoma is complete R0 resection, which usually requires major liver resection. The extent of biliary or vascular involvement can dictate the side of the liver that has to be resected. In other cases, a left- or right-sided liver resection can be chosen, which is a frequent subject of debate. Often, an extended right hepatectomy is advocated for the highest probability of R0 resection, however this is associated with a high risk of liver failure due to the small liver remnant.^1,2^

## Present

We performed a large retrospective study on 816 patients who underwent left-sided liver resection and 855 patients who underwent right-sided liver resection for perihilar cholangiocarcinoma.^3^ The 90-day mortality rate after right-sided resection was twice that after left-sided resection (18% vs. 9%; *p* < 0.001). Positive resection margins were similar for both groups but median overall survival was 23 months after right-sided liver resection and 30 months after left-sided liver resection (*p* < 0.001).

## Future

This study is the largest to date on this subject and highlights that a left-sided resection strategy is better tolerated and is associated with better survival. This study contradicts that extended right hepatectomy should be preferentially performed for perihilar cholangiocarcinoma. It also highlights that the perioperative risks of perihilar cholangiocarcinoma surgery are among the highest of any elective cancer surgery. Modifiable risk factors to decrease liver failure after extended right hepatectomy include better preoperative biliary drainage with less preoperative cholangitis, and liberal use of portal/hepatic vein embolization.^4,5^ A prospective study including all surgical decision-making details might be essential to finally settle the debate on the optimal surgical approach.
